# Concurrent nicotine exposure to prenatal alcohol consumption alters the hippocampal and cortical neurotoxicity

**DOI:** 10.1016/j.heliyon.2019.e03045

**Published:** 2020-01-08

**Authors:** Dwipayan Bhattacharya, Ayaka Fujihashi, Mohammed Majrashi, Jenna Bloemer, Subhrajit Bhattacharya, Manal Buabeid, Martha Escobar, Timothy Moore, Vishnu Suppiramaniam, Muralikrishnan Dhanasekaran

**Affiliations:** a Department of Drug Discovery and Development, Harrison School of Pharmacy, Auburn University, Auburn, AL, 36849, USA; b Department of Pharmacology, Faculty of Medicine, University of Jeddah, Jeddah, 23881, Saudi Arabia; c Department of Psychology, Auburn University, Auburn, Alabama, United States of America

**Keywords:** Neuroscience, Aging, Mitochondrial function, Oxidative stress

## Abstract

**Aims:**

This study investigated the neurotoxic effects of prenatal alcohol and nicotine exposure in the cortex and hippocampus of rodents.

**Main methods:**

Behavioral alterations, electrophysiological changes, and biochemical markers associated with cholinergic neurotransmission, neural oxidative stress, mitochondrial function, and apoptosis were evaluated.

**Key findings:**

Prenatal alcohol exposure induced the generation of ROS, nitrite and lipid peroxide, decreased mitochondrial Complex-I and IV activities, increased Caspase-1 and 3 activities, had no effect on cholinergic neurotransmission, increased expression of PSD-95, decreased LTP and decreased performance on spatial memory tasks. However, nicotine exposure, in addition to alcohol exposure, was found to mitigate the negative effects of alcohol alone on ROS generation and spatial memory task performances. Furthermore, we also studied the role of ILK in prenatal alcohol and nicotine exposure.

**Significance:**

Prenatal Smoking and/or drinking is a major health concern around the world. Thus, our current study may lead to better insights into the molecular mechanisms of fetal alcohol and nicotine exposure on the developing offspring.

## 1 Introduction

A growing concern in developed countries is drinking during pregnancy. A large number of women (around 15%) have admitted to have consumed alcohol at some stage of their pregnancy. A significant number of such women are heavy drinkers [1]. It is well known that drinking during pregnancy can damage the central nervous system (CNS) in the developing offspring impairing developmental and cognitive enrichments. Prenatal alcohol exposure can therefore cause some serious developmental problems collectively known as Fetal Alcohol Spectrum Disorders (FASD) [2]. Alcohol is a serious teratogen that can cause problems associated with the CNS, such as hyperactivity, poor movement coordination and muscle control, lower than average IQ, and cognitive deficits [3]. Some of these effects have been attributed to neuronal dysfunction in various regions of the brain of the offspring notably the hippocampus [4, 5], and children with FASD experience difficulty in spatial and memory tasks [6, 7]. Many animal models were used to study the effect of prenatal alcohol on overall brain development and memory. There was a significant reduction (20%) in the number of dorsal hippocampal neurons and long-term potentiation (LTP) in FASD rodent model. Like women consuming alcohol during pregnancy, there is a significant proportion of women (20–25%) who smoke nicotine during pregnancy [8]. Prenatal nicotine exposure augments the risk of sudden infant death syndrome (SIDS), low birthweight, and ectopic pregnancy [9, 10, 11]. Epidemiological reports indicate children exposed to nicotine during development have similar cognitive and behavioral problems as prenatal alcohol expsoure. The behavioral problems are hyperactivity and impulsivity, impaired learning and memory, and lower IQ. With respect to neuroanatomy, nicotine exposure during pregnancy leads to reduced cortical thickness and cell packing density, as well as altered neuronal morphology [12]. Clinical and experimental trials have indicated a strong association between nicotine and alcohol use [13], pointing to their concurrent usage. Thus, it appears that a significant population of women who consumes both these teratogens during pregnancy, yet a few clinical data exist on the cumulative effects of alcohol and nicotine on developing offspring [14]. As such, the interactive role of prenatal alcohol and nicotine on fetal brain development is not well understood. Some studies have shown deleterious effect of prenatal alcohol and nicotine on developing brain growth spurt and neuroanatomical organization of the brain [15]. In this study, we assessed the effect of prenatal alcohol and nicotine on neuronal stress, underlying signaling mechanisms and behavior in rat model. We specifically focused on the cortex and hippocampus of the during postnatal days of the offspring. The study was aimed to understand the effect of oxidative stress in these brain regions and their effect on synaptic receptor expression and electrophysiological output. Our previous reports identified Integrin Linked Kinase (ILK) as a potential signaling molecule that can alter synaptic receptor expression and Long Term Potentiation in FASD rat model [16]. We also looked into some of these mechanisms in the present alcohol + nicotine model.

## 2 Materials and methods

### 2.1 Animals

Auburn University Institutional Animal Care and Use Committee's (IACUC) approval was obtained for this study. Sprague Dawley (Time pregnant) rats were purchased from Charles River Laboratories (Wilmington, MA) and under isoflurane anesthesia the osmotic mini pumps (Alzet, Cupertino, CA) were implanted to deliver subcutaneous nicotine as per the previous study [17].

The various groups used in this study:

(i) Control: Water was administrated orally and subcutaneous saline via mini pump
(ii) Alcohol: Alcohol (10% v/v) mixed with water was administrated orally and subcutaneous saline via mini pump
(iii) Alcohol + nicotine: Alcohol (10% v/v) mixed with water was administrated orally and subcutaneous nicotine dissolved in sterile saline was used to deliver 6 mg/kg/day via mini pump

After birth, offsprings (5 male/group) were used to elucidate the neurotoxic effects of nicotine in prenatal alcohol exposure in the cortex and hippocampus. The offspring were sacrificed six weeks postnatally. The doses for the present study was based on our publication [16] and other previous studies [17, 18]. The dams consumed around 20 ml of alcohol solution (equivalent to 6 g/kg/day) which is comparable to 1–2 drinks per day for a human adult [19]. The consumption rate of 6 mg/kg/day is the most commonly studied dose for prenatal nicotine exposure in rats. This is thought to give plasma levels comparable to moderate to heavy smokers [19]. Alcohol was administered during dark cycle, because the animals are more active during this period, but was replaced with water during the light cycle to prevent dehydration. Food was administered ad libitum. The pumps were removed immediately after birth. Animals were housed at 12 h:12 h light:dark cycle (lights on at 6:00 am) and at a temperature of 22–24 °C. Pregnant dams were housed individually where as the pups were housed three per cage. Carbon dioxide based euthanasia was used in the current study.

### 2.2 Chemicals

Chemicals were purchased from Sigma (St. Louis, MO).

### 2.3 Biochemical studies

To avoid diurnal variations of biogenic molecules (endogenous amines, enzymes, and other antioxidant molecules), control, alcohol, and alcohol + nicotine exposed animals were sacrificed in the morning. The cortex and hippocampus was dissected out, immediately flash frozen in liquid nitrogen, and stored at − 80 °C. The cortical homogenate for the biochemical tests was prepared by homogenizing the tissue in 0.1 M phosphate buffer (pH 7.8), using a glass-Teflon homogenizer, followed by centrifugation at 10,000*g* for 60 min at 4 °C and the supernatant was isolated [20].

### 2.4 Assessment of reactive oxygen species (ROS)

Generation of reactive oxygen species (ROS): Spectrofluorometric method was used to determine ROS in the cortex of the control, alcohol and alcohol + nicotine treated animals. The ROS generated was measured at 492 nm (excitation) and 527 nm (emission). ROS (fluorescence units) measured was normalized to total protein content as relative fluorescence intensity/mg protein. The results are expressed as (%) change as compared to the control [21, 22].

### 2.5 Nitrite content

Nitrite content in the control, alcohol, and alcohol + nicotine treated rats were measured using Griess reagent at 545 nm [22].

### 2.6 Assessment of lipid peroxidation

Spectrophotometric method using thiobarbituric acid was used to assess lipid peroxidation in the cortex of the control, alcohol, and alcohol + nicotine treated animals. Lipid peroxidation was estimated by the formation of thiobarbituric acid-reactive substances (TBARS) at 532 nm. TBARS was normalized to total protein content as TBARS formed/mg protein. The results are expressed as (%) change as compared to the control [21, 22].

### 2.7 Glutathione (GSH) content quantification

Spectrofluorometric method (327 nm excitation and 423 nm emission) was used to determine GSH using o-phthalaldehyde (OPT). GSH measured was normalized to total protein content and reported as relative GSH content (μM)/mg protein [22].

### 2.8 Glutathione peroxidase activity

Spectrophotometric method was used to measure glutathione peroxidase activity in the cortex of the control, alcohol and alcohol + nicotine treated animals. The glutathione peroxidase activity was expressed as NADPH oxidized/mg total protein [22].

### 2.9 Superoxide dismutase activity (SOD)

Spectrophotometric method using pyrogallol was used to measure superoxide dismutase activity in the cortex of the control, alcohol, and alcohol + nicotine treated animals. The superoxide dismutase activity refers to inhibition of pyrogallol autoxidation/mg total protein [22].

### 2.10 Catalase activity

Spectrophotometric method using hydrogen peroxide as a substrate was used to measure catalase activity (240 nm) in the cortex of the control, alcohol, and alcohol + nicotine treated animals. The catalase activity refers to hydrogen peroxide oxidized/mg total protein [22].

### 2.11 Monoamine oxidase (MAO) activity

Spectrofluorometric method using kynuramine as a substrate was used to measure MAO activity (315 nm excitation and 380 nm emission) in the cortex of the control, alcohol and alcohol + nicotine treated animals. MAO activity refers to 4-hydroxy quinolone (μM)/formed/mg protein [20, 22, 23].

### 2.12 Complex-I activity

Spectrophotometric method using NADH as a substrate was used to measure Complex-I activity (340 nm) in the cortex of the control, alcohol and alcohol + nicotine treated animals. The Complex-I activity refers to NADH oxidized/mg protein [22, 23].

### 2.13 Complex-IV activity

Spectrophotometric method using cytochrome *c* as a substrate was used to measure Complex-IV activity (550 nm) in the cortex of the control, alcohol and alcohol + nicotine treated animals. The Complex-IV activity refers to cytochrome *c* oxidized/mg protein [22, 23].

### 2.14 Caspase-1 activity

Spectrofluorometric method using Ac-Tyr-Val-Ala-Asp-7-amino-4-Trifluoromethlcoumarin (Ac-YVAD-AMC) as a substrate was used to measure Caspase-1 (3260nm excitation and 460nm emission) activity (in the cortex of the Control, alcohol and alcohol + nicotine treated animals. The catalase activity refers to free AMC/mg total protein [22, 24].

### 2.15 Caspase-3 activity

Spectrofluorometric method using N-Acetyl-Asp-Glu-Val-Asp-7-amido-4-Methylcoumarin (Ac-DEVD-AMC) as a substrate was used to measure Caspase-3 (3260nm excitation and 460nm emission) activity in the cortex of the Control, alcohol and alcohol + nicotine treated animals. The catalase activity refers to free AMC/mg total protein [22, 24].

### 2.16 Choline acetyltransferase (ChAT) activity

Spectrophotometric method using choline chloride as a substrate was used to measure choline acetyltransferase activity (324nm) in the cortex of the control, alcohol and alcohol + nicotine treated animals. The ChAT activity refers to amount of 4-thiopyridone formed/mg protein. 4-thiopyridone (4-TP) is the product formed when reduced CoA reacts with 4,4′-dithiopyrdine (4-PDS) [25].

### 2.17 Acetylcholinesterase (AChE) activity

Spectrophotometric method using acetylthiocholine and 5,5′-dithio-bis-2-nitrobenzoic acid (DTNB) as substrates was used to measure acetylcholinesterase activity (412nm) in the cortex of the control, alcohol and alcohol + nicotine treated animals. The AChE activity refers to the amount of 5-thio-2-nitrobenzoate formed/mg protein. 5-thio-2-nitrobenzoate is the product formed when thiocholine—the product of the breakdown of acetylcholine—reacts with DTNB [26].

### 2.18 Western blot analysis

Total protein was isolated using cell lysis buffer (Cell Signaling Technology, Inc., Danvers, MA) containing protease inhibitor cocktail (P8340, Sigma, St. Louis, MO) and phosphatase inhibitors (P 5726, Sigma, St. Louis, MO). The expression of ILK was assessed using 1:1000 anti-ILK antibody (Cell Signaling)**.** β–actin was used as a loading control and was estimated using 1:1000 anti-β-actin antibody (Cell Signaling). 1:10000 anti-rabbit IgG, HRP-linked antibody was used as secondary antibody (Cell signaling). Band intensity was calculated by densitometric analysis using AlphaView software, normalized to β-actin, and reported as percentage change from the control. Protein concentration was measured using the Thermo Scientific Pierce 660 nm Protein Assay reagent kit (Pierce, Rockford, IL).

### 2.19 Immunoprecipitation

The brains from individual group of animals were pooled and PSD95 in addition to GluR2 were immunoprecipitated using 1:10 anti-PSD95 and anti- GluR2 antibodies respectively (Santa-Cruz) coated on Pure-Proteome A/G magnetic beads according to the manufacturer's protocol (Millipore). The beads containing the immunoprecipitated fraction was washed several times with 1X IMP buffer, pH = 7.4. Finally, 50ul of Laemmle buffer was added to the beads and boiled at 70 °C and proteins were detected using Western blot analysis.

### 2.20 Electrophysiology field recordings

Brains were isolated from euthanized rats and placed in ice-cold dissection solution containing (in mM) 120 NaCl, 11 D-Glucose, 26 NaHCO3, 6 MgCl2, 3 KCl, 0.5 CaCl2, 5 HEPES and 0.3 kynurenic acid. Coronal slices (350 uM) were made with a Leica VT-1200S. The slices were then transferred to artificial cerebrospinal fluid (aCSF, in mM 124 NaCl, 3 KCl, 1.5 MgSO47H20, 1.2 NaH2PO4, 2.4 CaCl2, 26 NaHCO3, and 10 D-Glucose bubbled with 95%CO2/5%O2) for at least 1 h and then maintained at room temperature until recording Electrophysiological recordings were performed as previously described (Bhattacharya 2015). Briefly, recordings were performed in a submerged chamber with continuous perfusion with aCSF (2–3 ml/min) bubbled with 95%CO2/5%O2 carbogen. fEPSPs were recorded from Schaffer collateral pathway SC-CA1 synapses with a glass pipette filled with aCSF (2–4MO). Stimulating pulses were applied at Schaffer collaterals via a bipolar stimulating electrode positioned 300 μm closer to CA3 subfield than the recording electrode. For LTP experiments, baseline was recorded at 50% of amplitude at which the initial population spike appeared. LTP was induced after 15 min of stable baseline recording using a Theta Burst Stimulation protocol (TBS), and recording was continued for 60 min post TBS. All electrophysiological data are presented as means ± SEM.

### 2.21 Behavior: Y maze

All animals were approximately 4 weeks old at the time of initiation of the study and there were no significant difference in the weight of the animals between each group (average weight was 57.8 g). Subjects were released into the Start arm and allowed to explore the Start and Other arm for 15 min, upon which time they were removed from the maze and returned to their home cages. Three hours later, all animals were returned to the maze and allowed to explore all three arms for 6 min. All scores were obtained from at least two independent, blinded observers. The following variables were recorded: (1) number of entries into each arm, and (2) dwell time into each arm. Measures for the Start and Other arm were averaged for all variables, and they will be referred as the Familiar arms. Dwell time was analyzed by contrasting the proportion of the total time spent in the maze's arms that subjects spent in the Novel and Familiar arms; these measures reflect exploratory behavior.

### 2.22 Contextual fear conditioning

Contextual fear conditioning was performed based on our previous publication on FASD [16].

### 2.23 other behavioral studies

Control, alcohol and alcohol + nicotine treated rats were observed by trained examiners. Straub tail, tremor and ataxic behaviors were monitored [27].

### 2.24 Protein quantification

Protein was quantified using Protein Assay reagent kit (Thermo Scientific Pierce 660 nm, Pierce, Rockford, IL). Bovine serum albumin (BSA) was used as a standard for protein measurement.

### 2.25 Statistical analysis

The results are presented as means ± SEM. Statistics were performed using the Prism-V software (La Jolla, CA, USA). The experimental data were analyzed using one-way analysis of variance (ANOVA) followed by Dunnet's multiple comparisons test. Statistical differences were considered significant at *p* < 0.05. Note (*) indicates a statistically significant difference when compared to controls.

## 3 Results

**Figure 1:**
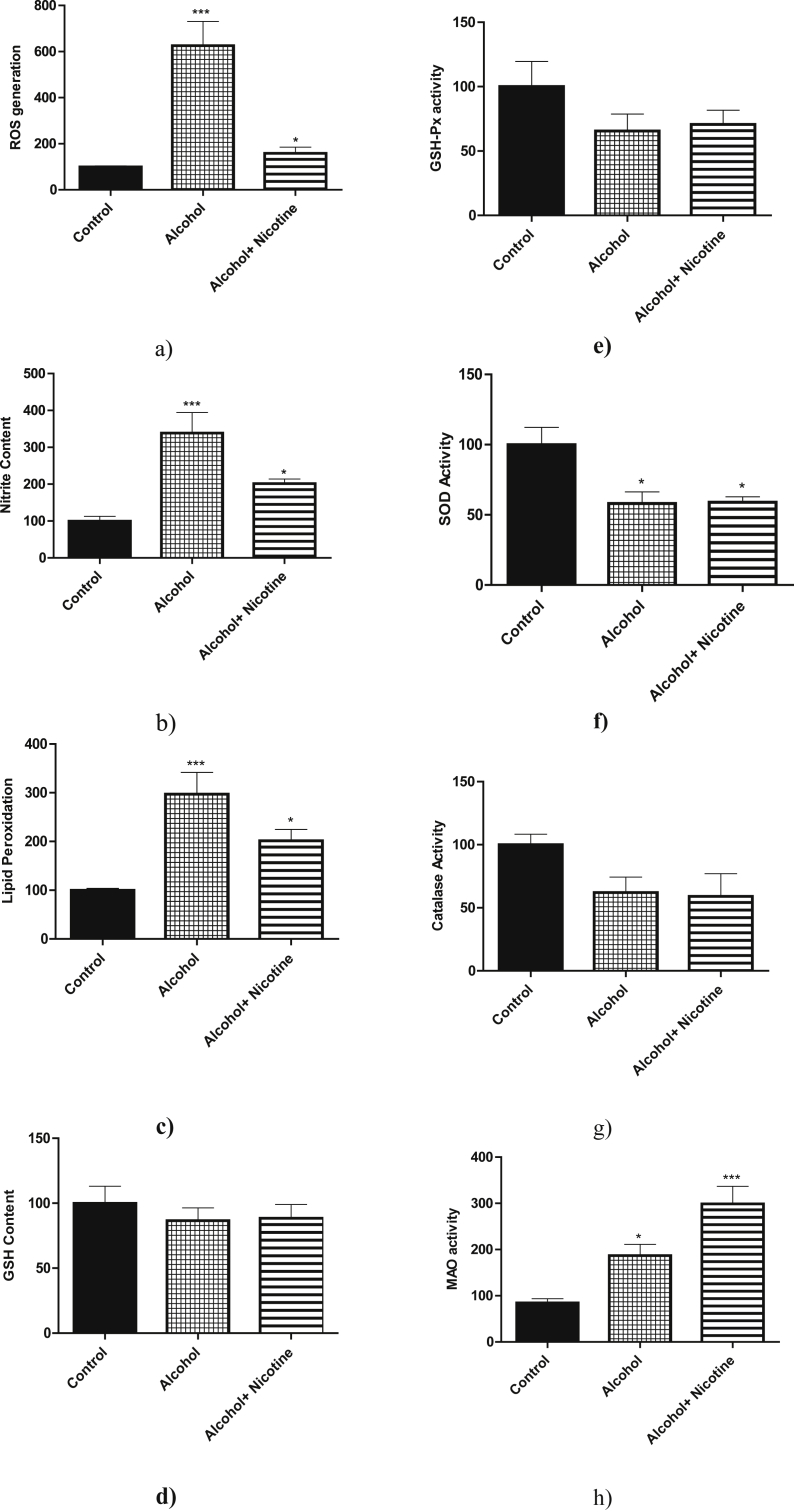
a: Effect of alcohol and alcohol + nicotine treatment on rat cortex reactive oxygen species generation: Reactive oxygen species was measured spectrofluorimetrically. Alcohol and alcohol + nicotine showed a significant increase in reactive oxygen species generation as compared to the control (*p < 0.05, n = 3). Reactive oxygen species generation was measured as relative fluorescence units (492/527 nm)/mg. Results are expressed as (%) change as compared to the control, Mean ± SEM. b: Effect of alcohol and alcohol + nicotine treatment on rat cortex nitrite content: Nitrite content was measured spectrophotometrically. Alcohol and alcohol + nicotine showed a significant increase in nitrite content as compared to the control (*p < 0.05, n = 5). Results are expressed as (%) change as compared to the control, Mean ± SEM. c: Effect of alcohol and alcohol + nicotine treatment on rat cortex lipid peroxide formation: Lipid peroxide was measured spectrophotometrically as thiobarbituric acid reactive substances (TBARS). Due to the increased reactive oxygen species generation and Nitrite content of alcohol and alcohol + nicotine induced a significant formation of lipid peroxide (*p < 0.05, n = 5). Lipid peroxide formation was measured as TBARS formed (532 nm)/mg protein. Results are expressed as (%) change as compared to the control ±SEM. d: Effect of alcohol and alcohol + nicotine treatment on rat cortex glutathione content: Glutathione content was measured spectrophotometrically. Alcohol and alcohol + nicotine had no significant effect on GSH content (n = 5). Results are expressed as (%) change as compared to the control, Mean ± SEM. e: Effect of alcohol and alcohol + nicotine treatment on rat cortex glutathione peroxidase activity: Glutathione peroxidase activity was measured spectrophotometrically using NADPH as substrate. Alcohol and alcohol + nicotine had no significant effect on glutathione peroxidase activity (n = 5). Results are expressed as (%) change as compared to the control, Mean ± SEM. f: Effect of alcohol and alcohol + nicotine treatment on rat cortex superoxide dismutase activity: Superoxide dismutase activity was measured colorimetrically using pyrogallol as a substrate. Alcohol and alcohol + nicotine showed a significant reduction in superoxide dismutase activity (*p < 0.05, n = 5). Results are expressed as (%) change as compared to the control, Mean ± SEM. g: Effect of alcohol and alcohol + nicotine treatment on rat cortex catalase activity: Catalase activity was measured spectrophotometrically using hydrogen peroxide as substrate. Alcohol and alcohol + nicotine had no significant effect on catalase activity (n = 5). Results are expressed as (%) change as compared to the control, Mean ± SEM. h: Effect of alcohol and alcohol + nicotine treatment on rat cortex monoamine oxidase activity: Monoamine oxidase activity was measured spectrofluorimetrically using kynuramine as substrate. Alcohol and alcohol + nicotine significantly increased monoamine oxidase activity (*p < 0.05, n = 5). Results are expressed as (%) change as compared to the control, Mean ± SEM.

Alcohol and alcohol + nicotine exposure increased the generation of ROS significantly in the cortex compared to the control (Figure 1a, *p* < 0.05). Likewise, nitrite content significantly increased in the cortex after exposure to alcohol and alcohol + nicotine as compared to the control (Figure 1b, *p* < 0.05). Due to the increase in the generation of ROS and increased nitrite content, alcohol and alcohol + nicotine exposure significantly increased lipid peroxidation as compared to the control (Figure 1c, *p* < 0.05). There was no significant effect on glutathione content (Figure 1d). With regard to the other antioxidant enzymes such as glutathione peroxidase, superoxide dismutase and catalase, prenatal exposure to alcohol and alcohol + nicotine did not affect the glutathione peroxidase (Figure 1e), but there was a significant decrease in superoxide dismutase activity of both experimental groups compared to the control (Figure 1f). There was no significant difference in the catalase activity among the groups (Figure 1g). Prenatal exposure to alcohol and alcohol + nicotine significantly increased the monoamine oxidase activity as compared to the control (Figure 1h).

**Figure 2:**
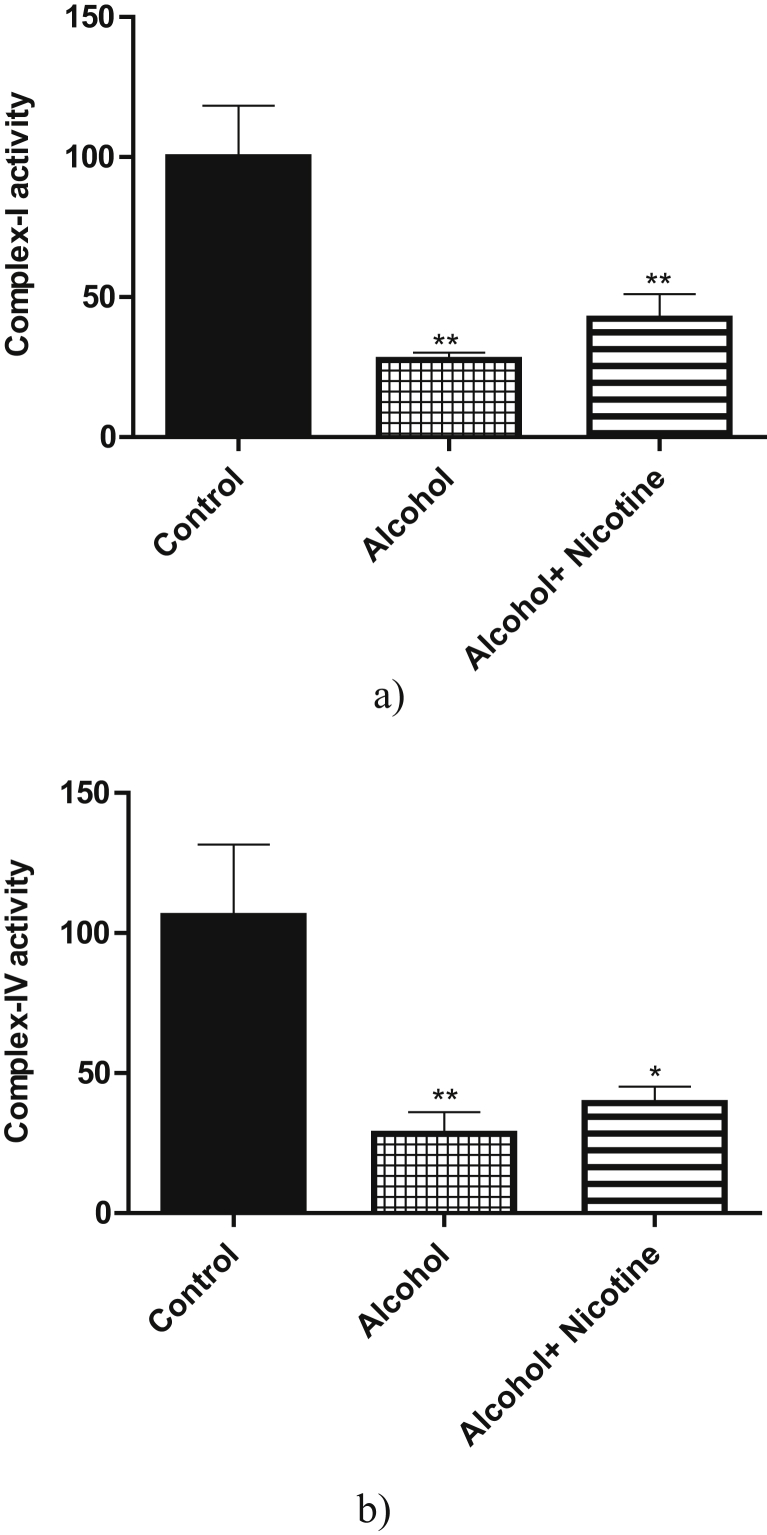
a: Effect of alcohol and alcohol + nicotine treatment on rat cortex Mitochondrial Complex-I activity: Mitochondrial Complex-I activity was measured spectrophotometrically using NADH as substrate. Alcohol and Alcohol + Nicotine showed a significant decrease in Mitochondrial Complex-I activity (*p < 0.05, n = 5). Results are expressed as (%) change as compared to the control, Mean ± SEM. b: Effect of alcohol and alcohol + nicotine treatment on rat cortex Mitochondrial Complex-IV activity: Mitochondrial Complex-IV activity was measured colorimetrically using Cytochrome-C as substrate. Alcohol and alcohol + nicotine showed a significant decrease in Mitochondrial Complex-IV activity (*p < 0.05, n = 5. Results are expressed as (%) change as compared to the control, Mean ± SEM.

**Figure 3:**
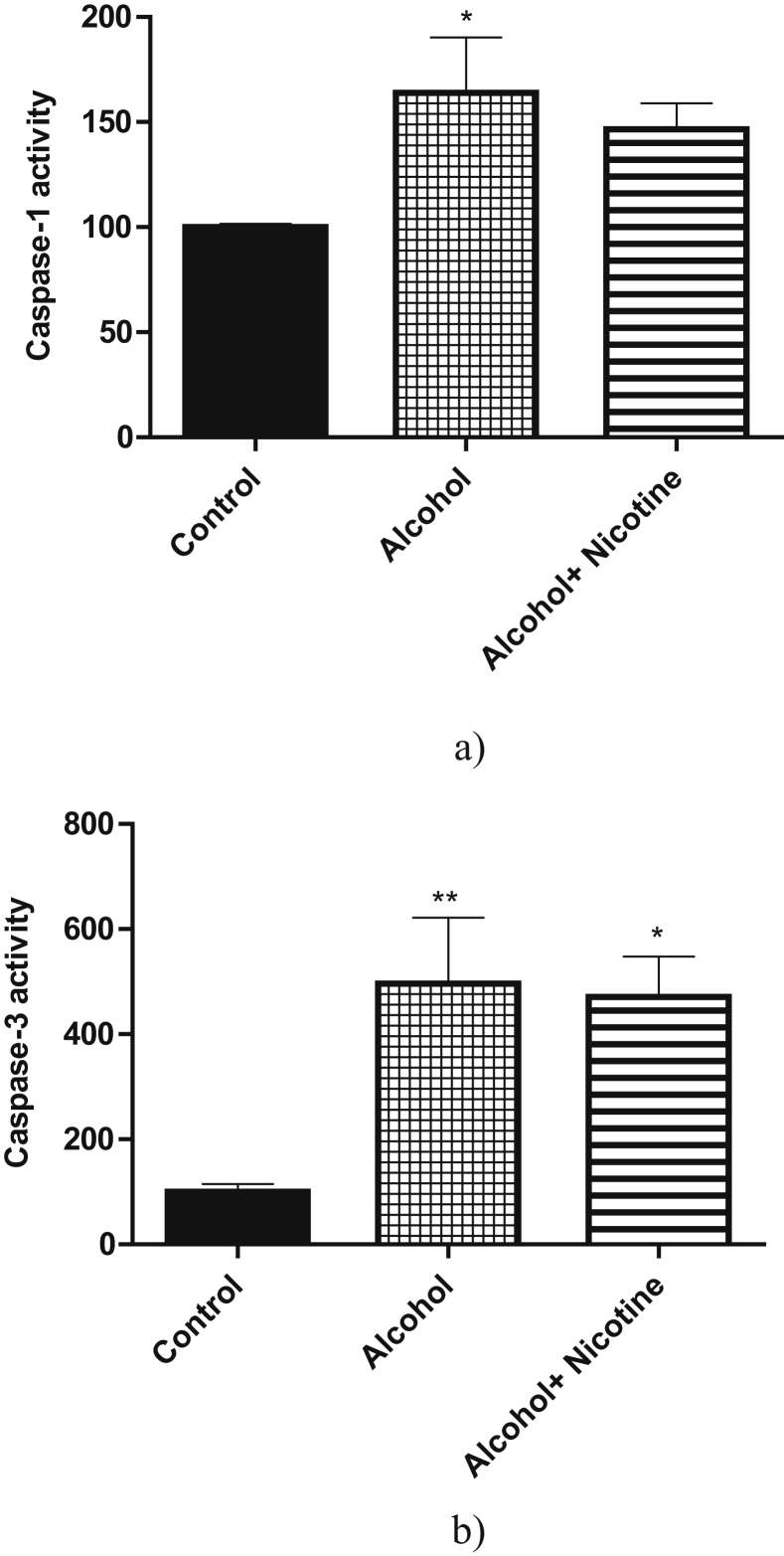
a: Effect of alcohol and alcohol + nicotine treatment on rat cortex Caspase-1 activity: Caspase-1 activity was measured spectrofluorimetrically using AC-YVAD-AMC as substrate. Alcohol significantly increased Caspase-1 activity (*p < 0.05, n = 5). Results are expressed as (%) change as compared to the control, Mean ± SEM. b: Effect of alcohol and alcohol + nicotine treatment on rat cortex Caspase-3 activity: Caspase-3 activity was measured spectrofluorimetrically using AC-DEVD-AMC as substrate. Alcohol and alcohol + nicotine significantly increased Caspase-3 activity (*p < 0.05, n = 5). Results are expressed as (%) change as compared to the control, Mean ± SEM.

**Figure 4:**
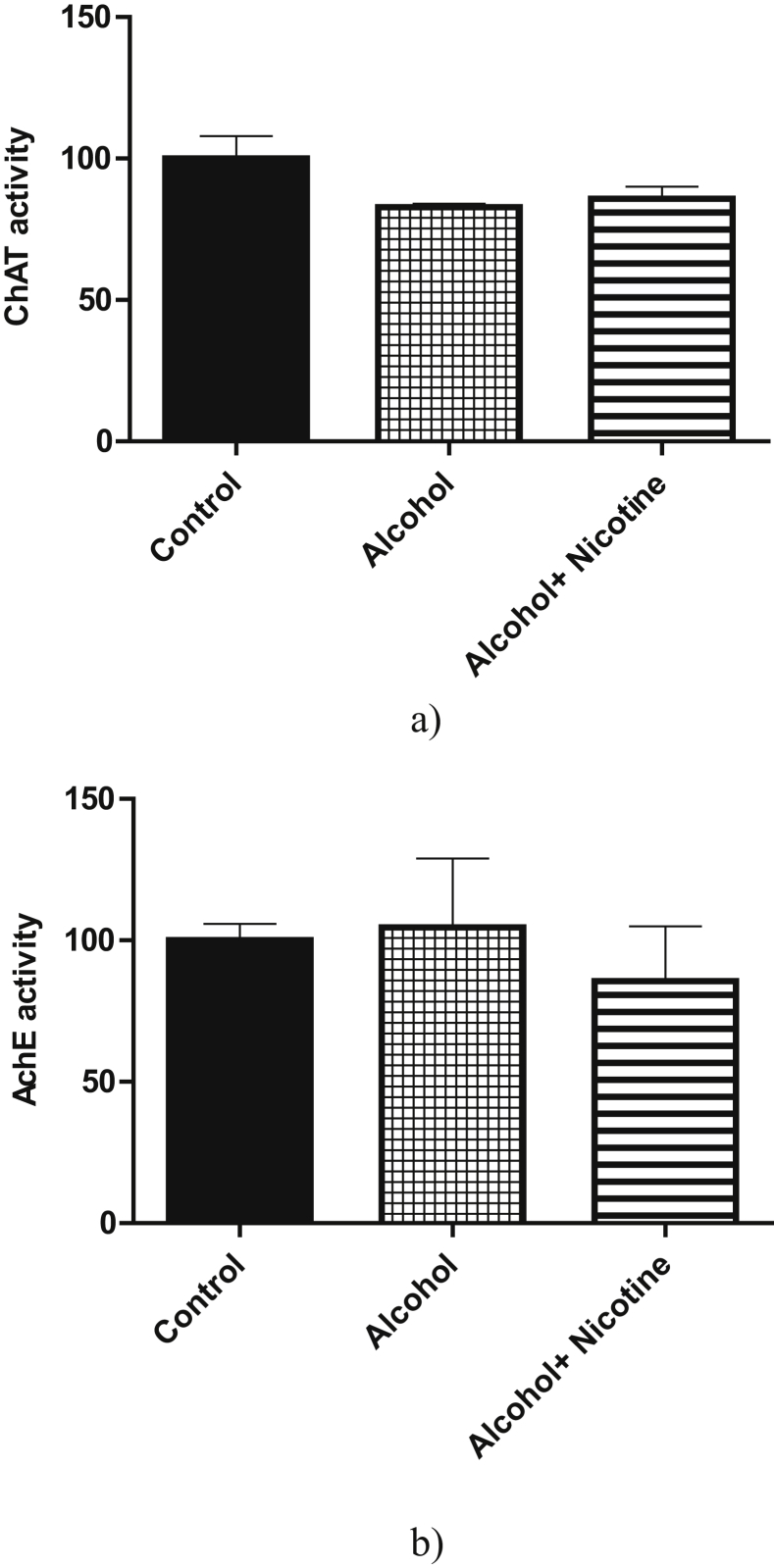
a: Effect of alcohol and alcohol + nicotine treatment on rat cortex acetylcholinesterase activity: Acetylcholinesterase activity was measured spectrofluorimetrically. Alcohol and alcohol + nicotine had no significant effect on acetylcholinesterase activity (n = 5). Results are expressed as (%) change as compared to the control, Mean ± SEM. b: Effect of alcohol and alcohol + nicotine treatment on rat cortex choline acetyltransferase activity: Choline acetyltransferase activity was measured spectrofluorimetrically. Alcohol and alcohol + nicotine had no significant effect on choline acetyltransferase activity (n = 5). Results are expressed as (%) change as compared to the control, Mean ± SEM.

With reference to the mitochondrial functions, prenatal exposure to alcohol and alcohol + nicotine caused significant deficits in both Complex-I and Complex-IV activity compared to the control (Figures 2a, 2b). Alcohol exposure caused a significant increase in both Caspase-1 and Caspase-3 activity as compared to the control. Alcohol + nicotine caused a significant increase in Caspase-3 activity only as compared to control (Figures 3a, 3b). There was no significant change in the acetylcholinesterase activity among the three groups (Figure 4a). Similarly, the three groups did not have a significant change in choline acetyltransferase activity (Figure 4b).

**Figure 5:**
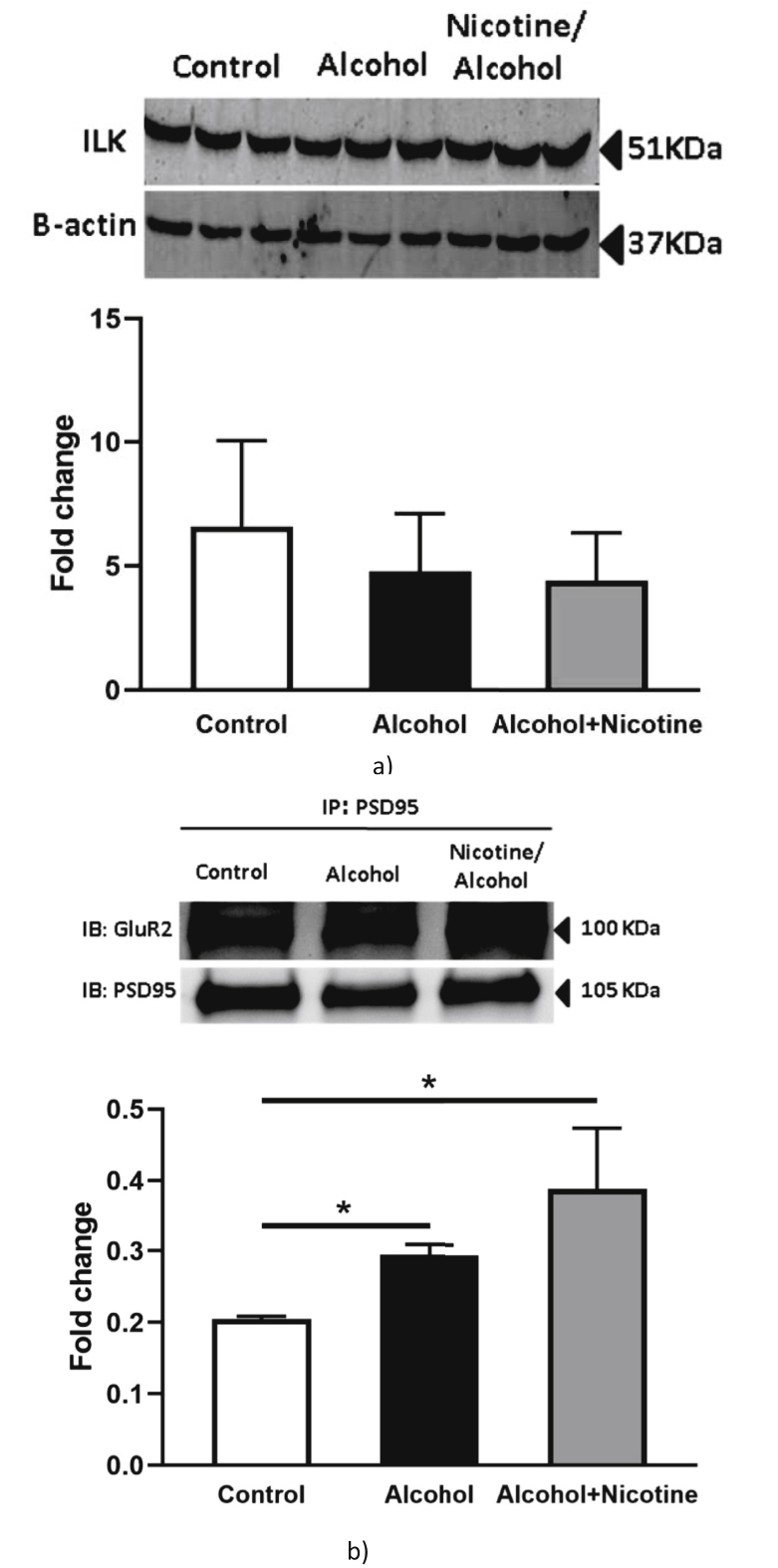
a: Effect of alcohol and alcohol + nicotine treatment on rat hippocampal protein expression (ILK): Alcohol and alcohol + nicotine had no effect on ILK expression (n = 5). Blots were developed using 1:1000 dilution with primary antibody. â-Actin (1:1000) was used as a loading control. Densitometric analysis was performed with AlphaView software. Results are expressed as (%) change as compared to the control, Mean ± SEM. b: Effect of alcohol and alcohol + nicotine treatment on rat hippocampal protein expression (GluR2 and PSD95): Prenatal alcohol and nicotine + alcohol exposure significantly increased the expression of GLuR2 (*p < 0.05, n = 5). Blots were developed using 1:1000 dilution with primary antibody. â-Actin (1:1000) was used as a loading control. Densitometric analysis was performed with AlphaView software and results are expressed as (%) control ±SEM. Please see supplementarysection for the full blot image.

**Figure 6:**
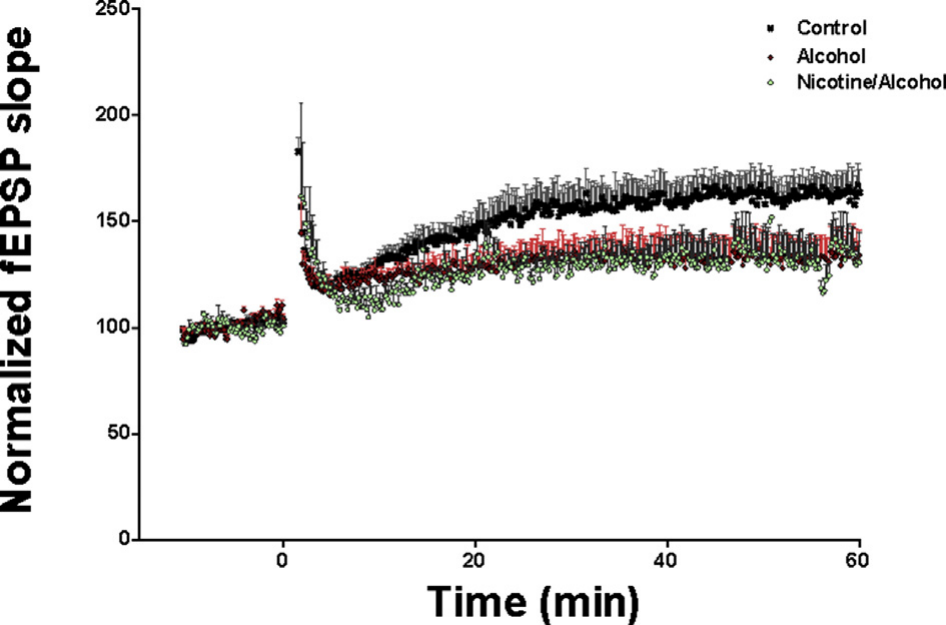
Effect of alcohol and alcohol + nicotine treatment on long term potentiation (LTP): Alcohol and alcohol + nicotine exposed animals showed significant deficits in LTP compared to control. However, there is no significant difference between the alcohol group and the alcohol + nicotine group.

The increase in ROS activity can alter the synaptic AMPA receptor expression and other synaptic proteins directly involving the mechanism of synaptic plasticity and cognitive behavior [28]. Activity dependent changes in LTP are the electrophysiological signature of changes in synaptic protein expression. We previously reported that ILK activity is involved in changes in the synaptic proteins in prenatal alcohol exposure model [16]. Hence, we looked into the neurotoxic mechanisms to further evaluate the effect of alcohol + nicotine co-exposure in the prenatal model. There is no significant change in expression of ILK among the three groups (Figure 5a). We reported earlier that ILK function change may affect the expression of synaptic AMPA receptors. We used scaffolding protein PSD95 pull down assay to look into the expression of GluR2 in total hippocampal protein lysate. There is almost 2-fold enhanced expression of GluR2 in the alcohol + nicotine group, whereas in the alcohol only group, the increase is around 1.5-fold as compared to the control group (Figure 5b) suggesting an additive effect on the synaptic AMPA receptor subtype expression. Increased GluR2 can result in reduced LTP and therefore we measured the electrophysiological field recording. In the hippocampal field recordings, alcohol and alcohol + nicotine showed a reduction in long-term potentiation following theta burst stimulation compared to controls, indicating alterations in synaptic plasticity. However surprisingly, we did not observe any additive effect of the two teratogens (Figure 6).

**Figure 7:**
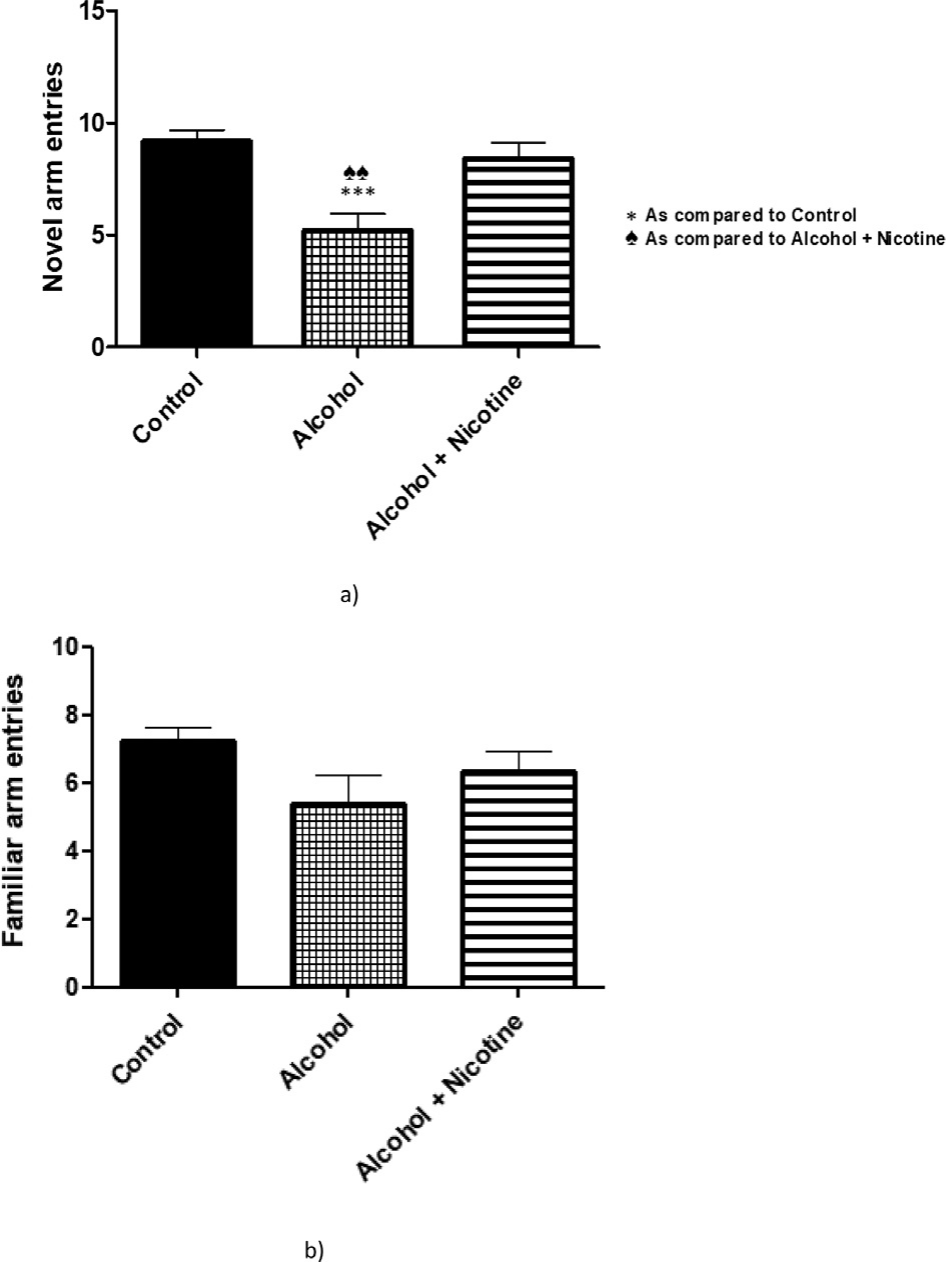
Effect of alcohol and alcohol + nicotine treatment on spatial memory tasks: Alcohol and alcohol + nicotine resulted in significant deficits in spatial memory tasks in the alcohol only treated group as compared to the control. Interestingly, the offspring exposed to prenatal nicotine and alcohol showed significant improvement in the spatial task as compared to the alcohol treatment. Bar chart shows the significant reduction in the number of entries by mice exposed to alcohol in exploring the novel arm in the Y-maze than the control mice and the alcohol + nicotine mice (*p < 0.05, n = 5).

We further looked into the general behavioral and specialized cognitive changes in the model. A battery of alcohol withdrawal related measurements was considered to assess the impact of teratogen co-exposure [29]. As expected, we did not notice any significant changes in straub tail, tremor and ataxic behaviors as compared to the controls (Table 1). There was no significant difference in the number of pups per litter or body weight observed among groups. Cognitive behavior task was also assessed in our model to understand the effect of these teratogens on rodent's usual exploratory behavior using Y-maze test. It mainly tests the willingness of a rodent to explore a novel arm as compared to the familiar arm which it has explored during training. If these teratogens affect the cognitive development associated with various brain regions, we may see an additive effect for both these teratogens. Unlike LTP data, we observed no significant change in spatial recognition and memory in prenatal alcohol + nicotine exposed rats. However, the prenatal alcohol exposed rats showed significant reduction in this spatial cognitive task (Figure 7a).

**Table 1:** Effect of alcohol and alcohol + nicotine on behavioral parameters.

Behavioral parameters	Control	Alcohol	Alcohol + Nicotine
Allergic reaction (redness of the Skin or eye)	N	N	N
Anaphylactic shock/Death	N	N	N
Ataxic behaviors	N	N	N
Diarrhea	N	N	N
Drooling	N	N	N
Fighting (aggressive Behavior)	N	N	N
Hair coat erection	N	N	N
Head twitching	N	N	N
Hind limb abduction	N	N	N
Hyperactivity (excessive Jumping)	N	N	N
Licking of the Genitals	N	N	N
Mortality observed	N	N	N
Penile erection (stimulatory Behavior)	N	N	N
Seizure	N	N	N
Straub tail	N	N	N
Tremor	N	N	N
Tumor	N	N	N

## 4 Discussion

Prenatal nicotine exposure has been associated with low birth weight, cognitive deficits in the offspring including low motor skills, hyperactivity, and learning disabilities and as well as an increased incidence of sudden infant death syndrome [14]. Maternal smoking induces oxidative stress in the offspring, as measured using F2-isoprostanes as the primary marker [15]. Mice embryo treated with nicotine exhibited an elevated Ca^2+^ concentration that is surmised to have led to an increase in the generation of reactive oxygen species and apoptosis [16]. Apoptotic effects of prenatal nicotine exposure have been supported by other studies in which nicotine exposure during pregnancy led to cell death in the midbrains and cerebral cortices of offspring [17]. Prenatal nicotine exposure in Wistar rats was also found to impair mitochondrial functions as seen by significantly reduced Complex-IV activity [16, 19].

Interestingly, similar developmental problems and toxic effects can be observed in prenatal alcohol exposure. Behavioral problems, such as aggression and poor social communication skills, deficits in intellectual functioning, namely, in short-term memory and encoding, reading & math skills, and overall mental processing can be observed in children prenatally exposed to alcohol. Human fetal brain cells have cytochrome P450 2E1, an enzyme that metabolizes alcohol into hydroxyl radicals that can further increase the generation of reactive oxygen species. Similarly, alcohol exposure during gestation in rodents has been shown to increase levels of oxidative stress in all organs, though the developing brain is most susceptible to oxidative stress-induced damage due to its high oxygen metabolic rate [30, 31]. Indeed, this generation of free radicals also affects mitochondrial functioning leading to reduced expression of Complex IV genes and ATP generation [24]. Alcohol exposure also increases caspase-3 activity and increased expression of pro-apoptotic genes leading to programmed cell death [16, 19].

Despite the mounting evidence on the harmful effects of alcohol and nicotine, some studies support the contrary. In fact, nicotine and its structural analogues have especially been of great interest as a potential therapeutic substance in treating Parkinson's disease [32]. In rat models of Parkinson's disease, nicotine infusions via a subcutaneous mini-osmotic pump stimulated the release of dopamine, leading to partial preservation of nigral dopamine neurons [33]. Indeed, epidemiological studies have found correlations between smoking and a lower incidence of Parkinson's disease [34]. Cigarette smoke exposure has also been found to mitigate the effects of MPTP [35]. Nicotine primarily acts on the nicotinic acetylcholine receptor (nAChRs), which can mediate protection against toxicity caused by glutamate, amyloid-β, and ethanol. Nicotine also exhibits antioxidant activity and reduces lipid peroxidation provoked by treatment with 6-hydroxydopamine [36]. Like nicotine, ethanol has also been suggested in potentially having a neuroprotective effect. Various epidemiological studies have found that moderate, non-binge consumers of alcohol had a significantly lower risk of cognitive loss or dementia [37]. However, some of the potential benefits of moderate alcohol consumption may be due to polyphenols found in alcohols such as wine [38]. Resveratrol, a polyphenol found in wine, is especially touted for its anti-inflammatory properties that have a cardioprotective effect by reducing infarct volume [39].

Various signaling processes are regulated by ILK and other growth factor signaling. β1 integrins in humans and rodents appear to be localized to ILK [40]. ILK plays a vital role in cancer research and facilitates various cellular functions such as survival, cytoskeletal dynamics, and proliferation [41]. ILK is required to promote neurite growth factor (NGF) mediated neurogenesis [42]. ILK interacts to AMPAR and PSD95 to modulate the effect of cocaine induced synaptic plasticity and memory [43]. ILK-related mechanisms are compromised in neurodegenerative diseases (e.g., Alzheimer's disease and Parkinson's disease) characterized by learning and memory deficits [44, 45]. We previously reported that alcohol exposure reduced ILK activity and also its interaction to GluR2 AMPA receptors [16, 19]. ILK inhibition could result in reduced phosphorylation of certain residues on GluR2 thereby increasing the stability of GluR2 at the surface. Increased expression of calcium impermeable AMPARs GluR2, would reduce the extent of depolarization at the synapse and therefore, would also contribute less to calcium influx thereby reducing LTP generation and maintenance [46]. However, prenatal alcohol + nicotine group failed to improve the synaptic plasticity deficits. The synaptic expression of increased GluR2 is not significantly reduced in alcohol + nicotine exposed animals. Moreover, the probability of GluR1 to GluR2 containing receptor switch responsible for LTP maintenance will be impaired with increased synaptic GluR2 [47]. This suggest that reduced plasticity in both alcohol and nicotine +alcohol group may be dependent on increased synaptic GluR2 expression. The complex interplay among the molecules GSK3β, BDNF, ILK and Akt strengthen the notion that ILK is critically involved in LTP. Analysis of basal synaptic transmission illustrates that ILK inhibition attenuates fEPSPs. These findings highlight a functional link between ILK, basal synaptic transmission and LTP in the hippocampus. Reduced LTP can also be due to increased GSK3β activation which could affect receptor trafficking and protein expression required for LTP maintenance in FASD model. Likewise, prenatal alcohol + nicotine group failed to improve the synaptic plasticity deficits as seen with FASD model. The enhanced GluR2 expression is one possible cause of plasticity deficits. Our previous study assessed the effects of prenatal exposure to ethanol on memory, using a contextual fear conditioning paradigm. This study revealed that prenatal alcohol exposure can cause the hippocampus related contextual memory deficit. Here, we tested the spatial memory task, a simpler way to understand the same complex brain development and its processing of spatial information. As expected, the prenatal alcohol exposure showed significant deficit in the memory development. Previous finding with prenatal nicotine only group also suggested impaired AMPA receptor function and LTP impairment as we see here in our co-administrative model. Therefore, we expected to see even stronger deficit in memory impairment with alcohol + nicotine exposure. On the contrary, we observed improved spatial task in our model of co-exposure as compared to control and alcohol only groups. This suggests nicotine can have a differential effect on memory development by antagonizing the deleterious effect of alcohol.

In summary, we demonstrated clearly that prenatal exposure to teratogens certainly has a significant change in neuronal circuit development involving the synaptic receptors and neurotransmitters. However, some protective effect with nicotine was observed in our cognitive task. This requires further exploration as various brain regions are involved in the exploratory behavior of these rodents. In this study we also compared the effects of alcohol + nicotine in the cortex and cerebellum (Table 2), however further specific behavioral tasks required to address the issue. More studies using specific pharmacological interventions are required to tease out the entire mechanism of synaptic regulation by these teratogens. The role of other AMPARs and NMDARs is to be assessed in order to understand the magnitude of neurotoxicity involved with these teratogens.

**Table 2:** Comparison of Markers of oxidative stress, mitochondrial functions, apoptosis and cellular signaling in Cortex and Cerebellum.

Marker	Cerebellum	Cortex
ILK	Significant decrease in both alcohol and alcohol + nicotine	No significant difference between three groups
PSD-95	Significant decrease in both alcohol and alcohol + nicotine, alc + nic decreased more than alcohol alone	Significant increase in both alcohol and alcohol + nicotine
COMPLEX-I	No significant difference between three groups	Significant decrease in both alcohol and alcohol + nicotine, alcohol decreased more than alc + nic
COMPLEX IV	No significant difference between three groups	Significant decrease in both alcohol and alcohol + nicotine, alcohol decreased more than alc + nic
CASPASE-3	Significant increase in both alcohol and alcohol + nicotine	Significant increase in both alcohol and alcohol + nicotine, alcohol is slightly higher than alcohol + nicotine
ROS	Significant increase in both alcohol and alcohol + nicotine	Significant increase in alcohol, slight, insignificant increase in alcohol + nicotine
LIPID PEROXIDATION	Significant increase in both alcohol and alcohol + nicotine, with no significant difference between the two experimental groups	Significant increase in both alcohol and alcohol + nicotine, alcohol greater than alc + nic

## 5 Conclusion

In our earlier studies, we have shown that the ILK pathway appears to play a significant role in memory and synaptic plasticity impairments in the prenatal alcohol exposed offspring(s). However, alcohol + nicotine did not affect ILK expression and also had less effect on the electrophysiological and behavioral changes. Based on the results obtained, at this particular dose and duration of prenatal alcohol + nicotine exposure has shown to reduce the neurotoxicity-induced by prenatal alcohol exposure alone. Further in-depth prenatal neurochemical studies exposing to various doses and duration should be conducted to understand the prenatal effects of prenatal alcohol + nicotine.

## Declarations

### Author contribution statement

Dwipayan Bhattacharya, Ayaka Fujihashi, Mohammed Majrashi, Jenna Bloemer, Subhrajit Bhattacharya, Manal Buabeid, Timothy Moore: Conceived and designed the experiments; Performed the experiments; Analyzed and interpreted the data.

Martha Escobar, Vishnu Suppiramaniam, Muralikrishnan Dhanasekaran: Conceived and designed the experiments; Contributed reagents, materials, analysis tools or data; Wrote the paper.

### Funding statement

This research did not receive any specific grant from funding agencies in the public, commercial, or not-for-profit sectors.

### Competing interest statement

The authors declare no conflict of interest.

### Additional information

No additional information is available for this paper.

## Appendix A Supplementary data

Blots.pdf Blots.pdf
