# Supplementary figures and images for: Concurrent nicotine exposure to prenatal alcohol consumption alters the hippocampal and cortical neurotoxicity
Source: Heliyon. 2020 Jan 8;6(1):e03045. doi: 10.1016/j.heliyon.2019.e03045 (PMC6953639; doi:10.1016/j.heliyon.2019.e03045)

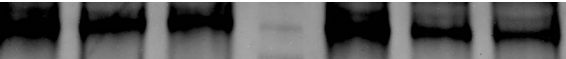

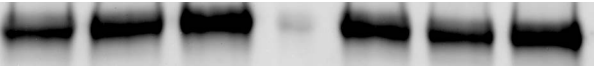

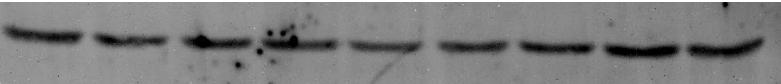

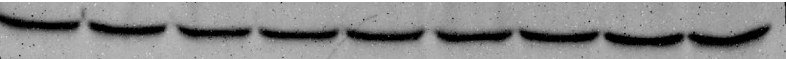

Supplement: Blots.pdf [file mmc1.pdf]
